# Neurological and Musculoskeletal Features of COVID-19: A Systematic Review and Meta-Analysis

**DOI:** 10.3389/fneur.2020.00687

**Published:** 2020-06-26

**Authors:** Auwal Abdullahi, Sevim Acaroz Candan, Muhammad Aliyu Abba, Auwal Hassan Bello, Mansour Abdullah Alshehri, Egwuonwu Afamefuna Victor, Naima Aliyu Umar, Burak Kundakci

**Affiliations:** ^1^Department of Physiotherapy, Bayero University, Kano, Nigeria; ^2^Department of Physiotherapy and Rehabilitation Sciences, University of Antwerp, Antwerp, Belgium; ^3^Department of Physiotherapy and Rehabilitation, Faculty of Health Sciences, Ordu University, Ordu, Turkey; ^4^Department of Physiotherapy, University of Ibadan, Ibadan, Nigeria; ^5^Department of Medical Rehabilitation, University of Maiduguri, Maiduguri, Nigeria; ^6^Physiotherapy Department, Faculty of Applied Medical Sciences, Umm Al-Qura University, Mecca, Saudi Arabia; ^7^NHMRC Center of Clinical Research Excellence in Spinal Pain, Injury and Health, School of Health and Rehabilitation Sciences, University of Queensland, Brisbane, QLD, Australia; ^8^Department of Medical Rehabilitation, Nnamdi Azikiwe University, Awka, Nigeria; ^9^Muhammad Abdullahi Wase Teaching Hospital, Kano, Nigeria; ^10^University of Nottingham, Academic Rheumatology, Nottingham, United Kingdom

**Keywords:** COVID-19, symptoms, myalgia, taste impairment, anosmia, cytokine storm, headache, muscle weakness

## Abstract

**Importance:** Some of the symptoms of COVID-19 are fever, cough, and breathing difficulty. However, the mechanism of the disease, including some of the symptoms such as the neurological and musculoskeletal symptoms, is still poorly understood.

**Objective:** The aim of this review is to summarize the evidence on the neurological and musculoskeletal symptoms of the disease. This may help with early diagnosis, prevention of disease spread, and treatment planning.

**Data Sources:** MEDLINE, EMBASE, Web of Science, and Google Scholar (first 100 hits) were searched until April 17, 2020. The key search terms used were “coronavirus” and “signs and symptoms.” Only studies written in English were included.

**Study Selection:** The selection was performed by two independent reviewers using EndNote and Rayyan software. Any disagreement was resolved by consensus or by a third reviewer.

**Data Extraction and Synthesis:** PRISMA guidelines were followed for abstracting data and assessing the quality of the studies. These were carried out by two and three independent reviewers, respectively. Any disagreement was resolved by consensus or by a third reviewer. The data were analyzed using qualitative synthesis and pooled using a random-effect model. Main Outcome(s) and Measure(s): The outcomes in the study include country, study design, participant details (sex, age, sample size), and neurological and musculoskeletal features.

**Result:** Sixty studies (*n* = 11, 069) were included in the review, and 51 studies were used in the meta-analysis. The median or mean age ranged from 24 to 95 years. The prevalence of neurological and musculoskeletal manifestations was 35% for smell impairment (95% CI 0–94%; *I*^2^ 99.63%), 33% for taste impairment (95% CI 0–91%; *I*^2^ 99.58%), 19% for myalgia (95% CI 16–23; *I*^2^ 95%), 12% for headache (95% CI 9–15; *I*^2^ 93.12%), 10% for back pain (95% CI 1–23%; *I*^2^ 80.20%), 10% for dizziness (95% CI 3–19%; *I*^2^ 86.74%), 3% for acute cerebrovascular disease (95% CI 1–5%; *I*^2^ 0%), and 2% for impaired consciousness (95% CI 1–2%; *I*^2^ 0%).

**Conclusion and Relevance:** Patients with COVID-19 present with neurological and musculoskeletal symptoms. Therefore, clinicians need to be vigilant in the diagnosis and treatment of these patients.

## Key Points

**Question**: What neurological and musculoskeletal symptoms of COVID-19 are reported in the literature, and what is their prevalence?
**Findings**: In this review, the reported neurological and musculoskeletal symptoms of COVID-19 are headache, dizziness, impaired consciousness, acute cerebrovascular disease, ataxia, seizure, impaired taste sensation, impaired smell sensation, impaired vision, myalgia, back pain, muscle weakness, skeletal muscle injury, arthralgia, and facial muscle pain. Their prevalence ranges from 1 to 35%.
**Meaning**: Patients with COVID-19 may present with symptoms such as anosmia, seizure, ataxia, and muscle weakness, which are not among the commonly reported symptoms (fever, cough, and breathing difficulty), and these are still not understood. Therefore, recognizing such symptoms may help in early diagnosis and prevention of the disease. Similarly, it will help with planning the treatment of such symptoms and prevention of further complications in the long term.

## Introduction

COVID-19 is the disease associated with a novel coronavirus strain (SARS-CoV-2) belonging to the Nidovirales order, a case of which was first reported in 2019 from Wuhan city in China (1). The disease is said to be transmitted through droplets from human saliva, eyes, and nose (2, 3). When humans come into contact with these droplets, the virus can get into the body through the same routes and lodge in the lungs (2). In the lungs, it will bind with the angiotensin-converting enzymes 2 (ACE 2) in the alveolar cells and destroy them (3, 4). The alveolar cells play important roles in human respiration (5), and their damage can impair the process of respiration. Since the functioning of other systems and organs of the body requires normal functioning of the respiratory system, its impairment will, in turn, impair the functions of those systems and organs, leading to a state of disequilibrium. Consequently, symptoms of COVID-19 can be many and may also vary. So far, the most common notable early symptoms of the disease are believed to be cough, headache, and fever (3). However, recently, evidence is emerging on the effect of COVID-19 on the nervous and musculoskeletal systems (6–8).

The effects of COVID-19 on the nervous and musculoskeletal systems may manifest as anosmia, olfactory function impairment, myalgia, muscle weakness, and Guillian Barre Syndrome (6–9). However, there is still little evidence on these, as scientists are still struggling to understand the disease process, including the pathogenicity, virual replication, and epidemiology (2, 7). Ironically, in some patients, some of these symptoms may precede the commonest symptoms of COVID-19 (10). In addition, symptoms such as myalgia, muscle weakness, and headache may render the patients unable to carry out activities of daily living (ADL) such as walking. In humans, the ability to carry out ADL is associated with good quality of life (11, 12). Furthermore, symptoms such as muscle weakness can result in complications such as muscle atrophy and contracture in the long term. Therefore, identifying the neurological and musculoskeletal features of the disease would be beneficial and can provide further information with which to understand the diagnosis of COVID-19 and how to manage patients. The aim of this review is to summarize the evidence on the neurological and musculoskeletal symptoms of COVID-19.

## Methods

### Design and Protocol

This is a systematic review and proportional meta-analysis that was conducted according to PRISMA (Preferred Reporting Items for Systematic Reviews and Meta-Analyses) guidelines (13). However, the protocol was not registered in any systematic review register because of the urgent need for literature on COVID-19 that can help curb the spread and impact of the disease.

### Eligibility Criteria and Information Sources

The inclusion criteria for this review were as follows: all studies with any study design that reported the neurological and musculoskeletal features in patients with COVID-19, studies published on or prior to April 17, 2020, and studies written in English. However, articles in the form of reviews, anecdotal description, and speculative considerations and editorials were excluded. Studies reporting exclusively on cases in children were also excluded. This is because the majority of the children infected with COVID-19 do not show any symptoms, and even in those who show symptoms, the symptoms tend to be limited to only mild fever and cough (14). Consequently, excluding children can enable us to generalize the findings to the adult population with COVID-19.

Four electronic databases, namely MEDLINE, EMBASE, Web of Science, and Google Scholar (first 100 hits), were searched from their date of establishment to April 17, 2020. The lists of references in the included studies were also screened for any relevant papers. The key search terms used were “coronavirus” and “signs and symptoms,” modified in terms of the glossary of each database and combined using Boolean operators. The search was carried out by one of the reviewers (BK). Appendix 1 demonstrates the search strategy applied in MEDLINE.

### Selection of Eligible Studies and Extraction of Data

EndNote and Rayyan were used to remove any duplicates and select eligible studies from the database findings and other sources (lists of references in included studies). Two independent reviewers (AA and SAC) who have experience in conducting systematic reviews selected the eligible studies using Rayyan software (15). Any disagreement between reviewers was resolved by consensus or by a third reviewer (BK).

A standardized form was used to extract the relevant data by three reviewers (AA, NUM, and MAA). The data extracted from each study were the study details (study title, first author, year, setting/country, study design), participant details (sex, age, overall sample size, number of patients in critical and non-critical conditions, comorbidities, diagnostic criteria used for the disease (COVID-19), and information about the treatments received), and neurological features, such as headache, dizziness, impaired consciousness, acute cerebrovascular disease, ataxia, seizure, taste impairment, smell impairment, vision impairment, neuropathic pain, and musculoskeletal features such as myalgia and back pain. In addition, the number of patients presenting with a particular symptom was also extracted.

### Assessment of the Methodological Quality of the Included Studies

A modified McMaster Critical Review Form for quantitative studies was used to critically assess the methodological quality of the included studies (16). This form is a comprehensive quality tool and can be used to assess all types of quantitative studies. It consists of 17 items with four answer options for each item (yes, no, not addressed and not applicable) to assess seven main components, including study purpose, literature review, study design, sample size, outcomes, interventions, results, and conclusions. Each item receives a score of zero when the answer to a particular item is no or not addressed and a score of one when the answer to a particular item is yes. However, when an item is not applicable to a particular study design, no score is awarded; NA is used to designate this. The scores from the tool are classified as poor, fair, good, or excellent, representing 1/4 or less, ≤2/4, ≥2/4 but ≤3/4, and >3/4 to 4/4 of the total score, respectively. The level of evidence was also determined using the National Health and Medical Research Council's (NHMRC) evidence hierarchy (17). Two independent reviewers performed the quality assessment (ABH and AA). Any disagreements between the first and the second reviewers were resolved through discussion to reach consensus and/or by a third reviewer (NUA). See the McMaster Critical Review Form in Appendix 2.

### Data Analysis

Qualitative and quantitative data (descriptive and proportional meta-analysis) analyses were performed. The descriptive analysis was performed and represented in the form of summary tables. The proportional meta-analysis was performed using StataSE 16 for the quantitative data if there were at least two studies that reported the proportion of the same clinical symptom. A random-effect model due to the heterogeneity and Freeman-Tukey double arc-sine transformation were used to stabilize the variance of specific prevalence rates to minimize the impact of studies with extremely small or extremely large prevalence estimates on overall estimates (18). The *I*^2^ index was also calculated to assess the level of heterogeneity, which can be classified into four categories: might not be important (0–40%), may represent moderate heterogeneity (30–60%), may represent substantial heterogeneity (50–90%), and considerable heterogeneity (75–100%). Publication bias was assessed using a funnel plot and Egger's test (19).

## Results

**Figure 1:**
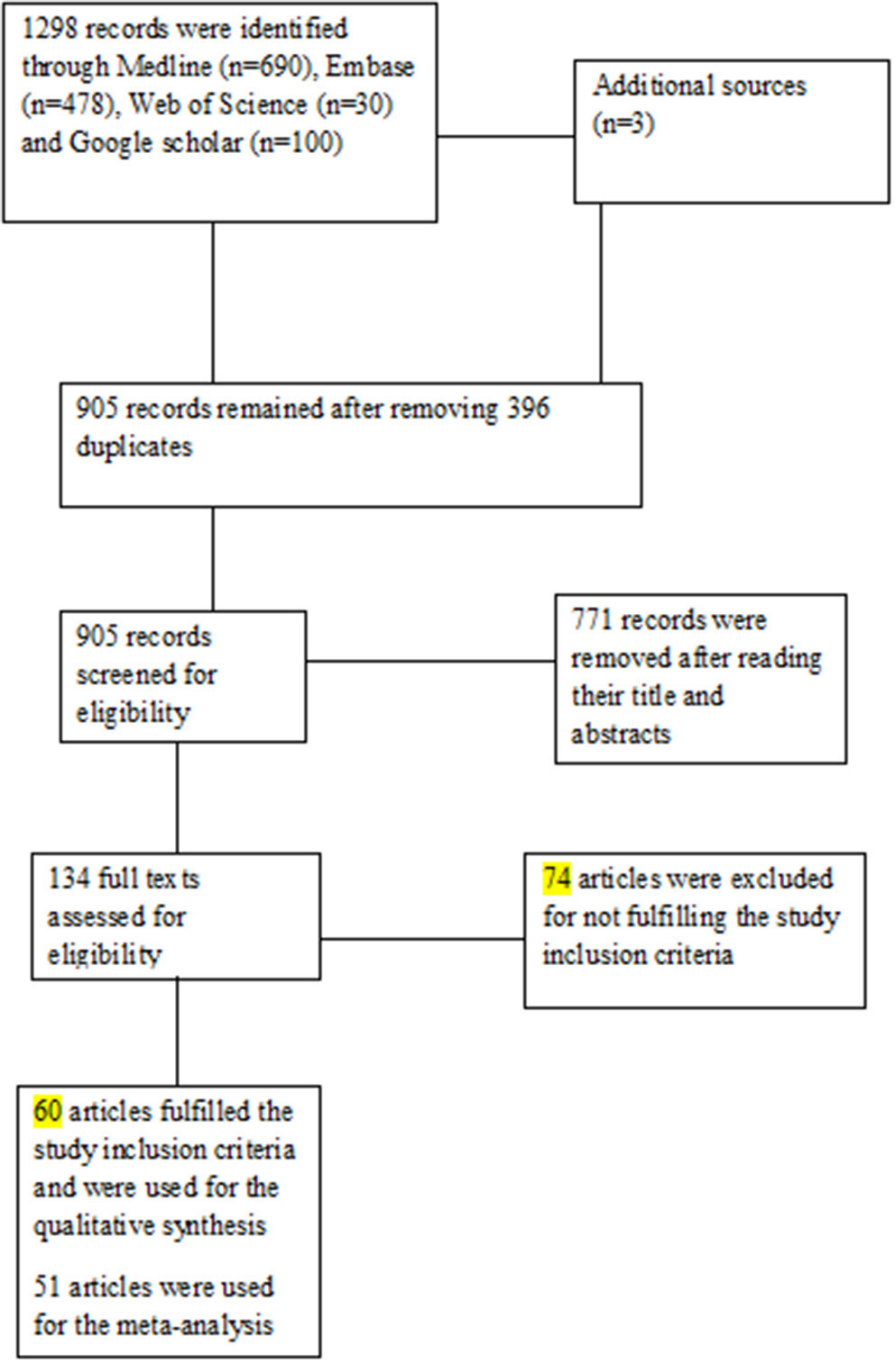
Study PRISMA Flow chart.

A total of 1,301 published articles were identified from the electronic databases (*n* = 1,298) and other sources (*n* = 3). After the removal of duplicate studies (*n* = 396), 905 studies were eligible for an initial screening based on titles and abstracts. Following the initial screening, 771 records were removed, and the full-texts of 134 articles were screened against the defined eligibility criteria. After the full-text screening, 60 articles (4, 9, 10, 20–76) met the inclusion criteria and were used for qualitative synthesis. For the quantitative synthesis, only 51 articles (4, 9, 20–29, 31, 32, 34–36, 38, 41–43, 45–70, 72, 73, 75, 76) were used. Figure 1 shows the PRISMA flowchart.

The total number of participants in the included studies was 11,069, of which 5,168 were male. The median or mean age of the participants ranges from 24 to 95 years. Based on the studies reporting the situations of the patients, there were 2,377 and 4,882 participants in critical and non-critical conditions, respectively. The most common neurological manifestation was headache (35 studies; 58.33%) (4, 23, 25–28, 30, 31, 34–36, 41–48, 50, 52, 53, 56–63, 67–70, 72), followed by dizziness (6 studies; 10%) (29, 34, 45, 53, 54, 56), impaired smell sensation (5 studies; 8.33%) (33, 34, 36, 43, 55), impaired taste sensation (4 studies; 6.67%) (34, 36, 43, 53), acute cerebrovascular disease (2 studies; 3.33%) (34, 57), ataxia (2 studies; 3.33%) (9, 34), seizure (2 studies; 3.33%) (30, 34), impaired consciousness (1 study; 1.6%) (30), and impaired vision (1 study; 1.6%) (34). However, one study (20) reported non-specified neurological symptoms. The most common musculoskeletal manifestation was myalgia (48 studies; 80%) (4, 20–23, 25–29, 31, 32, 36–38, 41–44, 46–71, 73, 75, 76), followed by back pain (4 studies; 6.67%) (25, 40, 62, 63), muscle weakness (1 study; 1.67%) (9), skeletal muscle injury (1 study; 1.67%) (34), arthralgia (1 study; 1.67%) (36), and facial muscle pain (1 study; 1.67%) (36).

In terms of the study design, four of the studies were case series (22, 34, 56, 76), 10 studies (9, 10, 27, 30, 33, 39, 40, 44, 71, 74) were case reports, and 46 studies (4, 20–26, 28–30, 33, 39, 40, 44, 71, 74) were either cohort or cross-sectional studies. All the studies were published in 2020. The settings/countries of the included studies were as follows: five studies (40, 43, 74–76) were carried out in the United States, and one each was carried out in the United Kingdom (33), Spain (38), Italy (9), South Korea (63), France (39), and Japan (30); one study (36) is a multicenter study carried out in Europe (Belgium, France, Italy, and Spain). The rest of the studies (4, 10, 20–29, 31–35, 37, 41–62, 64–68, 70–73) were carried out in China. In most of the studies, the Chinese national CDC recommended protocol, World Health Organization (WHO) interim guidance, and real-time polymerase chain reaction (RT-PCR) were used to confirm the diagnosis of the disease. There were many comorbidities in the included studies such as hypertension, diabetes, cardiac or cerebrovascular disease, malignancy, chronic kidney disease, pituitary adenoma, chronic obstructive pulmonary disease, chronic renal failure, and cancer. Others are pregnancy, hepatitis B infection, allergic rhinitis, immune-suppression, history of head trauma, and neurological disease. Table 1 shows the details and characteristics of the included studies.

**Table 1:** Details and characteristics of the included studies.

**References**	**Country**	**Study design**	**Number of patients (overall)**	**Number of patients (critical)**	**Number of patients (non-critical)**	**Number of male patients**	**Mean/median age (years)**	**Headache**	**Dizziness**	**Impaired consciousness**	**Acute cerebrovascular disease**	**Ataxia**	**Seizure**	**Taste impairment**	**Smell impairment**	**Vision impairment**	**Nerve pain**	**Myalgia**	**Back pain**	**Muscle weakness**	**Skeletal muscle injury**	**Arthralgia**	**Facial muscle pain**	**Illness onset**	**Neurological symptoms**
Feng et al. (20)	China	Cohort	476	124	352	271	53 (40–60)	NA	NA	NA	AA	NA	NA	NA	NA	NA	NA	55	NA	NA	NA			4 (2–7)	47/440 (10.7)
Lei et al. (21)	China	Cross-sectional	199	24	53	77	49.35	NA	NA	NA	NA	NA	NA	NA	NA	NA	NA	18	NA	NA	NA			NA	NA
Zhang et al. (22)	China	Cross-sectional	120	30	90	43	45.4	NA	NA	NA	NA	NA	NA	NA	NA	NA	NA	57	NA	NA	NA			NA	NA
Han et al. (23)	China	Cross-sectional	108	NA	NA	38	45	14	NA	NA	NA	NA	NA	NA	NA	NA	NA	12	NA	NA	NA			1-3(1)	NA
Qian et al. (25)	China	Cross-sectional	91	NA	NA	37	50 (IQR, 36.5 to 57.0)	7	NA	NA	NA	NA	NA	NA	NA	NA	NA	7	9	NA	NA			NA	NA
Chen et al. (26)	China	Cross-sectional	99	NA	NA	67	55·5 (13·1)	8	NA	NA	NA	NA	NA	NA	NA	NA	NA	11	NA	NA	NA			NA	NA
Jin et al. (27)	China	Cross-sectional	651	NA	NA	331	45.62	67	NA	NA	NA	NA	NA	NA	NA	NA	NA	71	NA	NA	NA			NA	NA
Zhang et al. (28)	China	Cross-sectional	645	NA	NA	328	40.78	67	NA	NA	NA	NA	NA	NA	NA	NA	NA	71	NA	NA	NA			NA	NA
Lon et al. (29)	China	Cross-sectional	10	4	6	3	54 (27–64)	NA	2	NA	NA	NA	NA	NA	NA	NA	NA	3	NA	NA	NA			NA	NA
Moriguchi et al. (30)	Japan	Case report	1	NA	NA	1	24	1	NA	1	NA	NA	1	NA	NA	NA	NA	NA	NA	NA	NA	NA	NA	NA	NA
Du et al. (31)	China	Cross-sectional	109	51	58	74	70.7	8	NA	NA	NA	NA	NA	NA	NA	NA	NA	19	NA	NA	NA	NA	NA	NA	NA
Zhang et al. (32)	China	Case series	5	NA	NA	4	45	NA	NA	NA	NA	NA	NA	NA	NA	NA	NA	3	NA	NA	NA	NA	NA	NA	NA
Gane et al. (33)	UK	Case report	1	NA	NA	1	48	NA	NA	NA	NA	NA	NA	NA	1		NA	NA	NA	NA	NA	NA	NA	NA	NA
Mao et al. (34)	China	Case series	214	58.2	58.2	87	131	28	36	16	6	1	1	12	11	3	NA	NA	NA	NA	23	NA	NA	NA	NA
Han et al. (35)	China	Cross-sectional	17	NA	NA	6	40	4	NA	NA	NA	NA	NA	NA	NA	NA	NA	NA	NA	NA	NA	NA	NA	NA	NA
Lechien et al. (36)	Multi-center/Europe	Cross-sectional	417	NA	NA	154	36.9 ± 11.4	188	NA	NA	NA	NA	NA	342	357	NA	NA	242	NA	NA	NA	133	Present	NA	NA
Jin and Tong (37)	China	Case report	1	NA	1	1	60	NA	NA	NA	NA	NA	NA	NA	NA	NA	NA	Present	NA	NA	NA	NA	NA	NA	NA
Barrasa et al. (38)	Spain	Cross-sectional	48	48	0	27	63.2 (12)	NA	NA	NA	NA	NA	NA	NA	NA	NA	NA	2	NA	NA	NA	NA	NA	NA	NA
Eliezer et al. (39)	France	Case report	1	0	1	0	40	NA	NA	NA	NA	NA	NA	NA	1	NA	NA	NA	NA	NA	NA	NA	NA	NA	NA
Zhao et al. (10)	China	Case report	1	0	1	0	61	NA	NA	NA	NA	NA	NA	NA	NA	NA	NA	NA	NA	NA	NA	NA	NA	NA	NA
Kim et al. (40)	USA	Case report	1	NA	1	1	42	NA	NA	NA	NA	NA	NA	NA	NA	NA	NA	NA	1	NA	NA	NA	NA	NA	NA
Lian et al. (41)	China	Restrospective study	788	78	710	407	54.72	75	NA	NA	NA	NA	NA	NA	NA	NA	NA	91	NA	NA	NA	NA	NA	NA	NA
Shi et al. (42)	China	Cohort study	416	NA	NA	205	64 (21–95)	9	NA	NA	NA	NA	NA	NA	NA	NA	NA	19	NA	NA	NA	AN	NA	NA	NA
Yan et al. (43)	USA	Cross sectional	59	NA	NA	29	18–79	25	NA	NA	NA	NA	NA	12	13	NA		20	NA	NA	NA	NA	NA	NA	NA
Yang et al. (44)	China	Case report	4	NA	NA	1	NA	2	NA	NA	NA	NA	NA	NA	NA		NA	1	NA	NA	NA	NA	NA	NA	NA
Mi et al. (45)	China	Restrospective study	10	NA	NA	2	34–87	1	3	NA	NA	NA	NA	NA	NA	NA	NA	NA	NA	NA	NA	NA	NA	NA	NA
Wu et al. (46)	China	Restrospective study	80	NA	NA	42	44 (11)	8	NA	NA	NA	NA	NA	NA	NA	NA	NA	13	NA	NA	NA	NA	NA	NA	NA
Lei et al. (47)	China	Restrospective study	14	NA	NA	8	12–83	2	NA	NA	NA	NA	NA	NA	NA	NA	NA	1	NA	NA	NA	NA	NA	NA	NA
Xu et al. (48)	China	Restrospective study	50	37	13	29	43.9 ± 16.8 (3–85)	5	NA	NA	NA	NA	NA	NA	NA	NA	NA	8	NA	NA	NA	NA	NA	NA	NA
LI et al. (49)	China	Restrospective study	25	16	9	12	48	NA	NA	NA	NA	NA	NA	NA	NA	NA	NA	17	NA	NA	NA	NA	NA	NA	NA
Yang et al. (50)	China	Retrospective multi-center cohort	149	NA	NA	81	45.11 ± 13.35	13	NA	NA	NA	NA	NA	NA	NA	NA	NA	5	NA	NA	NA	NA	NA	NA	NA
Chen et al. (51)	China	Retrospective study	9	NA	NA	0	26-40	NA	NA	NA	NA	NA	NA	NA	NA	NA	NA	3	NA	NA	NA	NA	NA	NA	NA
Liang et al. (52)	China	Retrospective study	1,590	1187	403	904	48.9 ± 16.3	205		20	NA	NA	NA	NA	NA	NA	NA	234	NA	NA	NA	NA	NA	NA	NA
Chen et al. (53)	China	Restrospective study	203	96	107	108	54 (20–91)	10	4	NA	NA	NA	NA	NA	NA	NA	NA	54	NA	NA	NA	NA	NA	NA	NA
Hu et al. (54)	China	Restrospective study	24	NA	NA	8	5-95		1	NA	NA	NA	NA	NA	NA	NA	NA	1	NA	NA	NA	NA	NA	NA	NA
Chu et al. (55)	China	Restrospective study	54	11	43	36	39 (26–73)		NA	NA	NA	NA	NA	NA	NA	NA	NA	3	NA	NA	NA	NA	NA	NA	NA
Wang et al. (56)	China	Case series	138	102	36	75	56 (42–68)	9	13	NA	NA	NA	NA	NA	NA	NA	NA	48	NA	NA	NA	NA	NA	NA	NA
Zheng et al. (57)	China	Retrospective analysis	161	30	131	80	45	12	NA	NA	4	NA	NA	NA	NA	NA	NA	18	NA	NA	NA	NA	NA	NA	NA
Guan et al. (58)	China	Cohort	1,099	173	926	64	47	150	NA	NA	NA	NA	NA	NA	NA	NA	NA	164	NA	NA	NA	NA	NA	4(2–7)	NA
Wu et al. (59)	China	Retrospective multicenter descriptive	80	3	77	39	46.1	13	NA	NA	NA	NA	NA	NA	NA	NA	NA	18	NA	NA	NA	NA	NA	NA	NA
Wang et al. (60)	China	Descriptive	1,012	0	1,012	524	50	152	NA	NA	NA	NA	NA	NA	NA	NA	NA	170	NA	NA	NA	NA	NA	NA	NA
Lui et al. (61)	China	Retrospective	137	NA	NA	61	57	13	NA	NA	NA	NA	NA	NA	NA	NA	NA	44	NA	NA	NA	NA	NA	NA	NA
Ye et al. (24)	China	Cohort	55	NA	NA	19	37	NA	NA	NA	NA	NA	NA	NA	NA	NA	NA	NA	NA	NA	NA	NA	NA	NA	NA
Yang et al. (62)	China	Retrospective	52	52	0	35	59·7	3	NA	NA	NA	NA	NA	NA	NA	NA	NA	6	1	NA	NA	NA	NA	NA	NA
Kim et al. (63)	South Korea	Cohort study	28	0	28	15	42.6	7	NA	NA	NA	NA	NA	NA	NA	NA	NA	7	7	NA	NA	NA	NA	NA	NA
Cheng et al. (64)	China	Cohort	11	NA	NA	8	50.36	NA	NA	NA	NA	NA	NA	NA	NA	NA	NA	3	NA	NA	NA	NA	NA	NA	NA
Xu et al. (65)	China	Cross sectional	51	NA	NA	25	42	NA	NA	NA	NA	NA	NA	NA	NA	NA	NA	8	NA	NA	NA	NA	NA	NA	NA
Cao et al. (66)	China	Cross sectional	102	NA	NA	53	45	NA	NA	NA	NA	NA	NA	NA	NA	NA	NA	35	NA	NA	NA	NA	NA	NA	NA
Wan et al. (67)	China	Cohort	135	56	40	95	49	34	NA	NA	NA	NA	NA	NA	NA	NA	NA	44	NA	NA	NA	NA	NA	NA	NA
Wang et al. (68)	China	Cross sectional	69	14	55	32	42	10	NA	NA	NA	NA	NA	NA	NA	NA	NA	21	NA	NA	NA	NA	NA	NA	NA
Du et al. (69)	China	Cross sectional	85	85	0	62	65.8	4		NA	NA	NA	NA	NA	NA	NA	NA	14	NA	NA	NA	NA	NA	NA	NA
Huang et al. (4)	China	Cross sectional	41	13	28	30	49·0	3	NA	NA	NA	NA	NA	NA	NA	NA	NA	18	NA	NA	NA	NA	NA	NA	NA
Xu et al. (70)	China	Cross sectional	62	NA	NA	35	41	21	NA	NA	NA	NA	NA	NA	NA	NA	NA	32	NA	NA	NA	NA	NA	NA	NA
Dongyan et al. (71)	China	Case report	1	0	1	1	35	NA	NA	NA	NA	NA	NA	NA	NA	NA	NA	1	NA	NA	NA	NA	NA	NA	NA
Wang et al. (72)	China	Cross sectional	339	NA	NA	166	69	12	NA	NA	NA	NA	NA	NA	AN	NA	NA	16	NA	NA	NA	NA	NA	NA	NA
Cai et al. (73)	China	Cross sectional	298	58	240	145	47.5	NA	NA	NA	NA	NA	NA	NA	NA	NA	NA	NA	NA	NA	NA	NA	NA	NA	NA
Tape et al. (74)	USA	Case report	1	NA	NA	0	79	NA	NA	1	NA	NA	NA	NA	NA	NA	NA	1	NA	NA	NA	NA	NA	NA	NA
Bhatraju et al. (75)	USA	Cross sectional	24	24	0	15	64	NA	NA	NA	NA	NA	NA	NA	NA	NA	NA	NA	NA	NA	NA	NA	NA	NA	NA
Goyal et al. (76)	USA	Case series	393	NA	NA	238	62.2	NA	NA	NA	NA	NA	NA	NA	NA	NA	NA	107	NA	NA	NA	NA	NA	NA	NA
Toscano et al. (9)	Italy	Case report	5	3	2	NA	NA	NA	NA	NA	NA	1	NA	NA	NA	NA	NA	NA	NA	4	NA	NA	NA	NA	NA

The methodological quality of the included studies was variable. Fifty-eight studies (4, 9, 10, 20–23, 25–39, 41–76) have excellent methodological quality, one study (40) has good methodological quality, and one study (24) has fair methodological quality. In terms of the level of the evidence (based on NHMRC evidence hierarchy), one study is a level II study, three studies are level III-I studies, 35 studies are level III-2 studies, seven studies are level III-3 studies, and 14 studies are level IV studies. Table 2 shows the methodological quality and the level of evidence of the included studies.

**Table 2:** Levels of evidence and methodological quality of the included studies.

**References**	**Design**	**Level of evidence**	**1**	**2**	**3**	**4**	**5**	**6**	**7**	**8**	**9**	**10**	**11**	**12**	**13**	**14**	**15**	**16**	**17**	**Total score**
Feng et al. (20)	Cohort	III-2	Yes	Yes	Yes	NA	NA	NA	NA	Yes	Yes	NA	NA	NA	Yes	Yes	Yes	NA	Yes	9/9
Lei et al. (21)	Cross-sectional	III-2	Yes	Yes	Yes	No	NA	NA	NA	Yes	Yes	NA	NA	NA	Yes	Yes	Yes	No	Yes	9/11
Zhang et al. (22)	Cross-sectional	III-2	Yes	Yes	Yes	NA	NA	NA	NA	Yes	Yes	NA	NA	NA	Yes	Yes	Yes	NA	Yes	9/9
Han et al. (23)	Cross-sectional	III-2	Yes	Yes	Yes	NA	NA	NA	NA	Yes	Yes	NA	NA	NA	No	Yes	Yes	NA	Yes	8/9
Qian et al. (25)	Cross-sectional	III-2	Yes	Yes	Yes	NA	NA	NA	NA	Yes	Yes	NA	NA	NA	Yes	Yes	Yes	NA	Yes	9/9
Chen et al. (26)	Cross-sectional	III-2	Yes	Yes	Yes	NA	NA	NA	NA	Yes	Yes	NA	NA	NA	No	No	Yes	NA	Yes	7/9
Jin et al. (27)	Cross-sectional	III-2	Yes	Yes	Yes	NA	NA	NA	NA	Yes	Yes	NA	NA	NA	No	No	Yes	NA	Yes	7/9
Zhang et al. (28)	Cross-sectional	III-2	Yes	Yes	Yes	NA	NA	NA	NA	Yes	Yes	NA	NA	NA	Yes	Yes	Yes	NA	Yes	9/9
Lon et al. (29)	Cross-sectional	III-2	Yes	Yes	Yes	NA	NA	NA	NA	Yes	Yes	NA	NA	NA	Yes	No	Yes	NA	Yes	8/9
Moriguchi et al. (30)	Case report	IV	Yes	Yes	Yes	NA	NA	NA	NA	Yes	Yes	NA	NA	NA	No	Yes	Yes	NA	Yes	8/9
Du et al. (31)	Cross-sectional	III-3	Yes	Yes	Yes	NA	NA	NA	NA	Yes	Yes	NA	NA	NA	No	Yes	Yes	NA	Yes	8/9
Zhang et al. (32)	Case series	IV	Yes	Yes	Yes	NA	NA	NA	NA	Yes	Yes	NA	NA	NA	No	Yes	Yes	NA	Yes	8/9
Gane et al. (33)	Case report	IV	Yes	Yes	Yes	NA	NA	NA	NA	Yes	Yes	NA	NA	NA	No	Yes	Yes	NA	Yes	8/9
Mao et al. (34)	Case series	IV	Yes	Yes	Yes	NA	NA	NA	NA	Yes	Yes	NA	NA	NA	Yes	Yes	Yes	NA	Yes	9/9
Han et al. (35)	Cross-sectional	III-2	Yes	Yes	Yes	NA	NA	NA	NA	Yes	Yes	NA	NA	NA	Yes	Yes	Yes	NA	Yes	9/9
Lechien et al. (36)	Cross-sectional	III-2	Yes	Yes	Yes	No	NA	NA	NA	Yes	Yes	NA	NA	NA	Yes	Yes	Yes	No	Yes	9/11
Jin and Tong (37)	Case report	IV	Yes	Yes	Yes	NA	NA	NA	NA	Yes	Yes	NA	NA	NA	Yes	Yes	Yes	NA	Yes	9/9
Barrasa et al. (38)	Cross-sectional	II	Yes	Yes	Yes	No	NA	NA	NA	Yes	Yes	NA	NA	NA	Yes	Yes	Yes	No	Yes	9/11
Eliezer et al. (39)	Case report	IV	Yes	Yes	Yes	NA	NA	NA	NA	Yes	Yes	NA	NA	NA	No	Yes	No	NA	Yes	7/9
Zhao et al. (10)	Case report	IV	Yes	Yes	Yes	NA	NA	NA	NA	Yes	Yes	NA	NA	NA	No	Yes	Yes	NA	Yes	8/9
Kim et al. (40)	Case report	IV	Yes	Yes	Yes	NA	NA	NA	NA	NA	NA	NA	No	NA	No	No	Yes	No	Yes	5/9
Lian et al. (41)	Retrospective study	III-3	Yes	Yes	Yes	NA	NA	NA	NA	Yes	Yes	NA	NA	NA	Yes	Yes	Yes	NA	Yes	9/9
Shi et al. (42)	Cohort	III-3	Yes	Yes	Yes	NA	NA	NA	NA	Yes	Yes	NA	NA	NA	Yes	Yes	Yes	NA	Yes	9/9
Yan et al. (43)	Cross sectional	III-1	Yes	Yes	Yes	No	NA	NA	NA	No	No	NA	Yes	NA	Yes	Yes	Yes	No	Yes	8/12
Yang et al. (44)	Case report	IV	Yes	Yes	Yes	NA	NA	NA	NA	Yes	Yes	NA	Yes	NA	No	NA	Yes	NA	Yes	8/9
Mi et al. (45)	Retrospective cohort study	III-3	Yes	Yes	Yes	NA	NA	NA	NA	Yes	Yes	NA	NA	NA	No	NA	Yes	Yes	Yes	8/9
Wu et al. (46)	Retrospective study	III-3	Yes	Yes	Yes	NA	NA	NA	NA	Yes	Yes	NA	NA	NA	Yes	Yes	Yes	NA	Yes	9/9
Lei et al. (47)	Retrospective study	III-2	Yes	Yes	Yes	NA	NA	NA	NA	Yes	Yes	NA	NA	NA	Yes	Yes	Yes	NA	Yes	9/9
Xu et al. (48)	Retrospective study	III-1	Yes	Yes	Yes	NA	NA	NA	NA	Yes	Yes	NA	NA	NA	Yes	Yes	Yes	NA	Yes	9/9
Li et al. (49)	Retrospective study	III-2	Yes	Yes	Yes	NA	NA	NA	NA	Yes	Yes	NA	NA	NA	Yes	Yes	Yes	NA	Yes	9/9
Yang et al. (50)	Retrospective cohort study	III-2	Yes	Yes	Yes	NA	NA	NA	NA	Yes	Yes	NA	NA	NA	Yes	Yes	Yes	NA	Yes	9/9
Chen et al. (51)	Retrospective study	III-3	Yes	Yes	Yes	NA	NA	NA	NA	Yes	Yes	NA	NA	NA	Yes	Yes	Yes	NA	Yes	9/9
Liang et al. (52)	Retrospective cohort study	III-2	Yes	Yes	Yes	NA	NA	NA	NA	Yes	Yes	NA	NA	NA	Yes	Yes	Yes	NA	Yes	9/9
Chen et al. (53)	Retrospective study	III-2	Yes	Yes	Yes	NA	NA	NA	NA	Yes	Yes	NA	NA	NA	NA	Yes	Yes	NA	Yes	9/9
Hu et al. (54)	Retrospective study	III-2	Yes	Yes	Yes	NA	NA	NA	NA	Yes	Yes	NA	NA	NA	Yes	Yes	Yes	NA	Yes	9/9
Chu et al. (55)	Retrospective study	III-2	Yes	Yes	Yes	NA	NA	NA	NA	Yes	Yes	NA	NA	NA	Yes	Yes	Yes	NA	Yes	9/9
Wang et al. (56)	Case series	IV	Yes	Yes	Yes	NA	NA	NA	NA	Yes	Yes	NA	NA	NA	Yes	Yes	Yes	NA	Yes	9/9
Zheng et al. (57)	Retrospective study	III-2	Yes	Yes	Yes	NA	NA	NA	NA	Yes	Yes	NA	NA	NA	Yes	Yes	Yes	NA	Yes	9/9
Guan et al. (58)	Cohort	III-2	Yes	Yes	Yes	NA	NA	NA	NA	Yes	Yes	NA	NA	NA	No	Yes	Yes	NA	Yes	8/9
Wu et al. (59)	Retrospective multi-center descriptive	III-2	Yes	Yes	Yes	NA	NA	NA	NA	Yes	Yes	NA	NA	NA	No	Yes	Yes	NA	Yes	8/9
Wang et al. (60)	Descriptive	III-2	Yes	Yes	Yes	NA	NA	NA	NA	Yes	Yes	NA	NA	NA	Yes	Yes	Yes	NA	Yes	9/9
Wang et al. (61)	Retrospective study	III-2	Yes	No	Yes	NA	NA	NA	NA	Yes	Yes	NA	NA	NA	No	Yes	Yes	NA	Yes	7/9
Ye et al. (24)	Cohort	III-2	Yes	No	No	NA	NA	NA	NA	Yes	Yes	NA	NA	NA	No	No	Yes	NA	Yes	5/9
Yang et al. (62)	Retrospective study	III-3	Yes	Yes	Yes	NA	NA	NA	NA	Yes	Yes	NA	NA	NA	Yes	Yes	Yes	NA	Yes	9/9
Kim et al. (63)	Cohort study	III-2	Yes	Yes	Yes	NA	NA	NA	NA	Yes	Yes	NA	NA	NA	No	No	Yes	NA	Yes	7/9
Cheng et al. (64)	Cohort	III-1	Yes	Yes	Yes	NA	NA	NA	NA	Yes	Yes	NA	NA	NA	Yes	Yes	Yes	NA	Yes	9/9
Xu et al. (65)	Cross-sectional	III-2	Yes	Yes	Yes	NA	NA	NA	NA	Yes	Yes	NA	NA	NA	Yes	Yes	Yes	NA	Yes	9/9
Cao et al. (66)	Cohort	III-2	Yes	Yes	Yes	No	NA	NA	NA	Yes	Yes	NA	NA	NA	Yes	Yes	Yes	NA	Yes	9/10
Wan et al. (67)	Cohort	III-2	Yes	Yes	Yes	No	NA	NA	NA	Yes	Yes	NA	NA	NA	Yes	Yes	Yes	NA	Yes	9/10
Wang et al. (68)	Cross-sectional	III-2	Yes	Yes	Yes	No	NA	NA	NA	Yes	Yes	NA	NA	NA	Yes	Yes	Yes	No	Yes	9/11
Du et al. (69)	Cross-sectional	III-2	Yes	Yes	Yes	NA	NA	NA	NA	Yes	Yes	NA	NA	NA	Yes	Yes	Yes	NA	Yes	9/9
Huang et al. (4)	Cross-sectional	III-2	Yes	Yes	Yes	No	NA	NA	NA	Yes	Yes	NA	NA	NA	Yes	Yes	Yes	NA	Yes	9/10
Xu et al. (70)	Cross-sectional	III-2	Yes	Yes	Yes	NA	NA	NA	NA	Yes	Yes	NA	NA	NA	No	Yes	Yes	NA	Yes	8/9
Dongyan et al. (71)	Case report	IV	Yes	Yes	Yes	NA	NA	NA	NA	Yes	Yes	NA	NA	NA	No	Yes	Yes	NA	Yes	8/9
Wang et al. (72)	Cross-sectional	III-2	Yes	Yes	Yes	NA	NA	NA	NA	Yes	Yes	NA	NA	NA	No	Yes	Yes	NA	Yes	8/9
Cai et al. (73)	Cross-sectional	III-2	Yes	Yes	Yes	No	NA	NA	NA	Yes	Yes	NA	NA	NA	No	Yes	Yes	No	Yes	8/11
Tape et al. (74)	Case report	IV	Yes	Yes	Yes	NA	NA	NA	NA	Yes	Yes	NA	NA	NA	No	Yes	Yes	NA	Yes	8/9
Bhatraju et al. (75)	Cross sectional	IV	Yes	Yes	Yes	NA	NA	NA	NA	Yes	Yes	NA	NA	NA	No	Yes	Yes	NA	Yes	8/9
Goyal et al. (76)	Retrospective case series	IV	Yes	Yes	Yes	NA	NA	NA	NA	Yes	Yes	NA	NA	NA	No	Yes	Yes	NA	Yes	8/9
Toscano et al. (9)	Case report	IV	Yes	No	Yes	NA	NA	NA	NA	Yes	Yes	NA	Yes	NA	No	NA	Yes	NA	Yes	7/9

**Figure 2:**
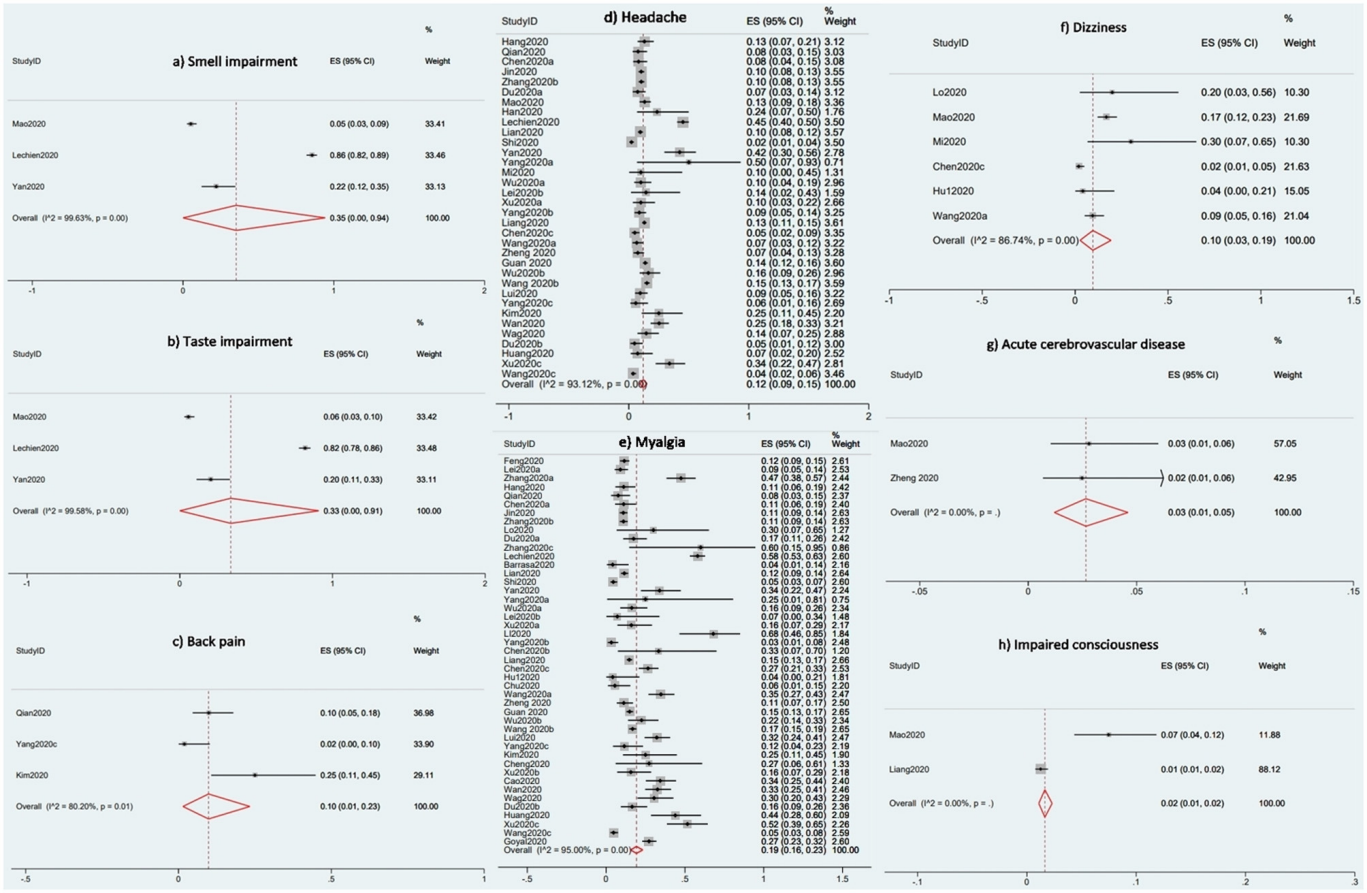
Forest plots for the prevalence of the Neurological and Musculoskeletal Features of Covid-19.

The proportional meta-analyses revealed that the prevalence of common neurological and musculoskeletal manifestations was 35% for smell impairment (95% CI 0–94%; *I*^2^ 99.63%), 33% for taste impairment (95% CI 0–91%; *I*^2^ 99. 58%), 19% for myalgia (95% CI 16–23; *I*^2^ 95%), 12% for headache (95% CI 9–15; *I*^2^ 93.12%), 10% for back pain (95% CI 1–23%; *I*^2^ 80.20%), 10% for dizziness (95% CI 3–19%; *I*^2^ 86.74%), 3% for acute cerebrovascular disease (95% CI 1–5%; *I*^2^ 0%), and 2% for impaired consciousness (95% CI 1–2%; *I*^2^ 0%). Figure 2 shows the forest plots for the prevalence of acute cerebrovascular disease, impaired consciousness, back pain, dizziness, headache, myalgia, smell impairment, and taste impairment.

**Figure 3:**
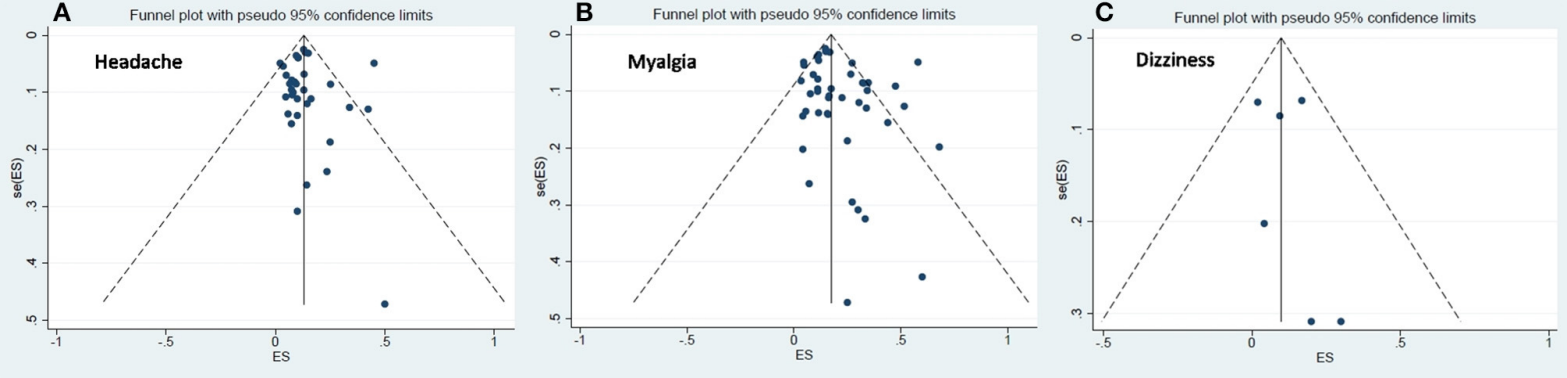
Funnel plots for publication bias for headache, dizziness and myalgia.

The visual symmetry of the funnel plots suggests that there was no publication bias for headache, dizziness, and myalgia. These results were also confirmed by Egger's test, which revealed statistically insignificant *p*-values. See Figure 3 for the funnel plots.

## Discussion

The results showed that the prevalence of neurological and musculoskeletal manifestations of COVID-19 was 35% for smell impairment, 33% for taste impairment, 19% for myalgia, 12% for headache, 10% for back pain, 3% for acute cerebrovascular disease, and 2% for impaired consciousness. In addition, the majority of the studies have excellent methodological quality, which is an indication of the validity and reliability of the studies (77). Thus, it is important that clinicians consider these symptoms during diagnosis of the disease and management of the patients to help prevent the spread of the disease and the development of any complications. For instance, acute cerebrovascular disease can manifest as symptoms such as stroke, seizure, and headache, which can result in long-term disability that may require rehabilitation for a very long time (78–80). Similarly, symptoms such as muscle weakness, myalgia, vision impairment, and arthralgia can interfere with patients' ability to carry out activities of daily living (ADL). When people are able to carry out ADL, they tend to have better quality of life (11, 12).

In addition, two of the most important factors about COVID-19 are that it is highly contagious and most of the people infected may not present with any notable symptoms such as fever and cough (56). This means that the presence of some previously unnoticed symptoms such as muscle weakness, visual impairment, and arthralgia may not raise any suspicion of the disease. As such, many unnoticed cases could infect many others and increase the spread of the disease. Delineating the whole spectrum of the symptoms patients with COVID-19 present with can help with prompt diagnosis, isolation, and treatment of cases.

One important finding in this study is that there are more neurological symptoms than musculoskeletal symptoms in patients with COVID-19. This may not be surprising, as the virus is believed to be neurotrophic, and the patients may therefore present with neurological symptoms or complications, especially in the long term (81, 82). Similarly, it is also possible that the patients will present with more musculoskeletal symptoms and complications in the long-term due to prolonged immobilization (83, 84). It is also worth noting that many of the symptoms in patients with COVID-19 are non-specific and cannot be highlighted as support for the early diagnosis of the disease. For instance, symptoms such as headache and impaired consciousness may be related to the respiratory failure. However, based on the reviewed studies reporting on the situations of the patients, the majority of the participants were not in critical condition. Therefore, it cannot be said with certainty that these non-specific symptoms are the result of respiratory failure.

Nevertheless, several factors may be the likely causes of the neurological and musculoskeletal features of COVID-19. Firstly, the virus may gain access to, for example, the central nervous system via the bloodstream and infect endothelial cells or leukocytes or through retrograde neuronal routes by infecting the peripheral nerves (85). Secondly, the virus causes pneumonia, which may result in systemic hypoxia, which will eventually damage the brain and other nerve cells (86). The processes through which the damage occurs include peripheral vasodilatation, hypercabia, hypoxia, and anaerobic metabolism, which ultimately result in neuronal swelling and brain edema (87). Neural swelling and brain edema can raise intracranial pressure and result in impaired consciousness and seizure or can irritate the trigeminal nerve and cause headache (88, 89). In addition, cytokine storms characterized by increased levels of inflammatory cytokines and activities of T lymphocytes, macrophages, and endothelial cells can also cause neuronal damage. In particular, the release of interleukin-6 causes vascular leakage and activation of complement and coagulation cascades (90). Consequently, it was noted that patients with the severe disease (COVID-19) tend to have higher levels of D-dimer, which is a marker of a hypercoagulable state and endogenous fibrinolysis (34, 91). These may be the factors that cause acute cerebrovascular disease in patients with COVID-19. Similarly, the elevated level of serum interleukin-6 during cytokine storms could be the cause of myalgia (92). The cytokine storm may also be the cause of the arthralgia presented by the patients. This is because interleukin-6 is a pro-inflammatory substance (93), and viral infections are also known to cause arthralgia (94). Thus, it is possible that arthralgia, which is joint pain, is associated with myalgia in patients with COVID-19.

Although we excluded studies in children because they generally present only with mild fever and cough (14), we recommend that they should be kept under close observation, since damage to the developing nervous system can be devastating. According to the World Health Organization (WHO), recent findings on symptoms in children testing positive for COVID-19 have shown unexplained inflammatory syndrome, mostly in several European and North American countries (95). However, due to the uncertainties of the definitions of symptoms associated with COVID-19 in children, it is important that more evidence is allowed to emerge before their presenting symptoms are categorized into definite neurological and/or musculoskeletal symptoms (95).

This review has multiple strengths, such as the estimation of prevalence for both neurological and musculoskeletal manifestations, the inclusion of a large number of studies (*n* = 60) with considerable sample size (*n* = 11, 069), the assessment of methodological quality and the level of evidence, and the use of proportional meta-analyses for the quantitative data. In addition, even though two systematic reviews on the neurological features of COVID-19 have been published previously (96, 97), this review seems to be the only one reporting symptoms such low-back and facial pain. Similarly, the study also has some limitations. One of the limitations is that the reviewed studies could not account for whether or not the neurological and musculoskeletal symptoms of COVID-19 are due to the comorbidities and/or the medicines the patients use for the comorbidities. This is because a number of comorbidities are reported in the studies, and possibly the comorbidities or the drugs the patients take may be responsible for one or more of these neurological or musculoskeletal symptoms. Other limitations are related to the search process, where gray literature databases were not searched and non-English language studies were not included. However, the lists of references of all included studies were screened to include all relevant studies and reduce the risk of publication bias. Furthermore, the heterogeneity between studies was high in most of the meta-analysis results. This may impact negatively on the certainty of the findings.

## Conclusions

Patients with COVID-19 present with many different symptoms, including those that affect the neurological and musculoskeletal systems. Therefore, delineating the whole spectrum of symptoms of the disease can help with early diagnosis, prevention of the spread of the disease, and its treatment. In addition, it will help with the prevention of complications that may arise in the long term.

## Data Availability Statement

The raw data supporting the conclusions of this article will be made available by the authors, without undue reservation.

## Author Contributions

AA conceived and designed (initially) the study and wrote up the results (with inputs from BK) and the discussion. SC, BK, NU, AB, EA, MAb, and MAl provided inputs to improve the design of the study. In particular, SC and BK modified the search strategy and the data extraction form. BK searched the literature. AA and SC selected the studies for eligibility. AA, NU, and MAb extracted the study data. AB, AA, and NU did the assessment of the methodological quality of the included studies. AA did the qualitative synthesis, and BK did the meta-analysis. AA and EA wrote up the introduction. MAl wrote up the methodology, which was modified by AA. SC, BK, EA, and MAl critically reviewed the manuscript. All authors contributed to the article and approved the submitted version.

## Conflict of Interest

The authors declare that the research was conducted in the absence of any commercial or financial relationships that could be construed as a potential conflict of interest.
