# Supplementary material for: Neurological and Musculoskeletal Features of COVID-19: A Systematic Review and Meta-Analysis
Source: Front Neurol. 2020 Jun 26;11:687. doi: 10.3389/fneur.2020.00687 (PMC7333777; doi:10.3389/fneur.2020.00687)
Supplement: Supplementary file 1 [file Data_Sheet_1.docx]

# **Appendices**

**Appendix 1**: Search Strategy in Medline

1. exp Coronavirus/

2. Coronavirus Infections/

3. covid 2019.mp.

4. SARS2.mp.

5. SARS-CoV-2.mp.

6. severe acute respiratory syndrome coronavirus 2.mp.

7. coronavirus infection.mp.

8. severe acute respiratory pneumonia outbreak.mp.

9. novel cov.mp.

10. 2019ncov.mp.

11. sars cov2.mp.

12. cov2.mp.

13. ncov.mp.

14. covid-19.mp.

15. covid19.mp.

16. Coronaviridae/

17. corona virus.mp.

18. 1 or 2 or 3 or 4 or 5 or 6 or 7 or 8 or 9 or 10 or 11 or 12 or 13 or 14 or 15 or 16 or 17

19. "Signs and Symptoms"/

20. (sign? adj2 symptom*).tw.

21. (sign? or symptom* or complain*).tw.

22. (clinical adj3 (manifestation? or feature? or finding? or aspect? or marker?)).tw.

23. (presenting adj3 (feature? or finding? or factor?)).tw.

24. presentation?.tw.

25. (physical adj3 (manifestation? or characteristic? or feature? or finding?)).tw.

26. 19 or 20 or 21 or 22 or 23 or 24 or 25

27. 18 and 26

28. limit 27 to yr="2019 -Current"

**Appendix 2**: Modified McMaster Critical Review Form for Quantitative Studies
